# Tris(μ_2_-2-meth­oxy-6-{[(2-sulfido­eth­yl)imino]meth­yl}phenolato)trinickel(II) di­methyl­formamide monosolvate: crystal structure, spectroscopic characterization and anti­bacterial activity

**DOI:** 10.1107/S2056989019004730

**Published:** 2019-04-12

**Authors:** Julia A. Rusanova, Vladimir N. Kokozay, Olena V. Pokas

**Affiliations:** a Department of Chemistry, Taras Shevchenko National University of Kyiv, 64/13, Volodymyrska str., Kyiv 01601, Ukraine; b Institute of Epidemiology and Infectious Diseases of the Academy of Medical, Sciences of Ukraine, 5, M. Amosova str., Kyiv 03038, Ukraine

**Keywords:** crystal structure, trinuclear Ni (II) complex, Schiff bases, cyste­amine (2-amino­ethane­thiol), *o*-vaniline, anti­bacterial activity

## Abstract

The title trinuclear nickel(II) complex, [Ni_3_(C_10_H_11_NO_2_S)_3_]·C_3_H_7_NO, with a Schiff base ligand formed *in situ* from 2-amino­ethane­thiol and *o*-vanillin crystallizes in the ortho­rhom­bic space group *Pbca*. Its asymmetric unit consists of one neutral Ni_3_ *L*_3_ mol­ecule and one DMF solvent mol­ecule. The solid-state organization of the complex can be described as an insertion of the solvent mol­ecules within the crystallographically independent trinuclear Ni^II^ species. Several C—H⋯π edge-to-face inter­actions and π–π stacking inter­actions link the components in the crystal. A first example of a short inter­molecular C—H⋯Ni contact is found. Anti­bacterial *in vitro* screening revealed that the title compound has anti­bacterial activity, the best effect being against *Acinetobacter baumannii*.

## Abstract 2

The crystal structure of a trinuclear Ni^II^ complex with a Schiff base ligand formed *in situ* from cyste­amine (2-amino­ethane­thiol) and *o*-vanillin is reported as well results of its anti­bacterial activity screening.

## Chemical context   

Schiff base ligands are one of the most widely utilized classes of ligands in metal coordination chemistry because of their preparative accessibility, structural variety and strong metal-binding ability with many metal ions *via* azomethine HC=N or phenolic groups (Garnovskii *et al.*, 1993 ▸; Bera *et al.*, 1998 ▸; Prabhakaran *et al.*, 2004 ▸). *o*-Vanillin-based Schiff ligands demonstrate an exceptionally rich coordination chemistry and diverse properties – magnetism, luminescence, chirality, catalysis, cytotoxicity and ferroelectricity (Andruh, 2015 ▸). The N and S atoms play a key role in the coordination of metals at the active sites of numerous metallobiomolecules. It has been shown that ONS Schiff bases are moderately active against leukemia (Tofazzal *et al.*, 2000 ▸). In particularly, nickel complexes with a multidentate NSO-containing mixed-ligand environment attract attention because such complexes play an important role in bioinorganic chemistry and redox enzyme systems and can be considered as model objects for studying the active sites of biological systems (Halcrow *et al.*, 1994 ▸). In this work we present the crystal structure of a novel trinuclear Ni^II^ complex with an NSO-type Schiff base ligand derived from *o*-vanillin and 2-amino­ethane­thiol as well results of its anti­bacterial activity screening against several Gram-positive and Gram-negative bacteria.

## Structural commentary   

**Figure 1:**
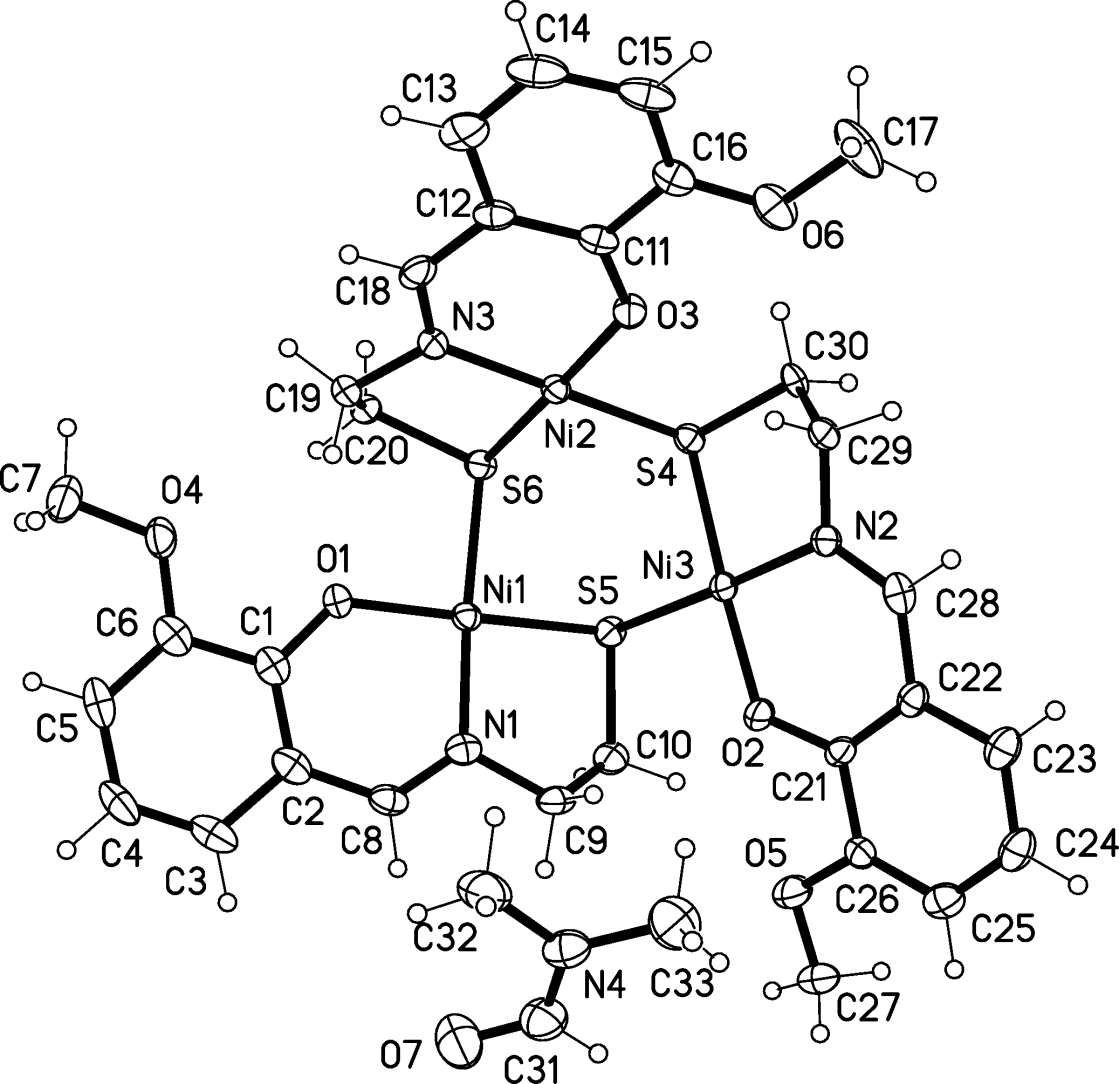
The mol­ecular structure of the trinuclear complex unit of the title compound, showing 50% probability displacement ellipsoids.

The title complex crystallizes in the ortho­rhom­bic space group *Pbca*. The asymmetric unit consists of one neutral Ni_3_ *L*_3_ mol­ecule and one DMF solvent mol­ecule. The mol­ecular structure of the trinuclear complex unit is depicted in Fig. 1 ▸.
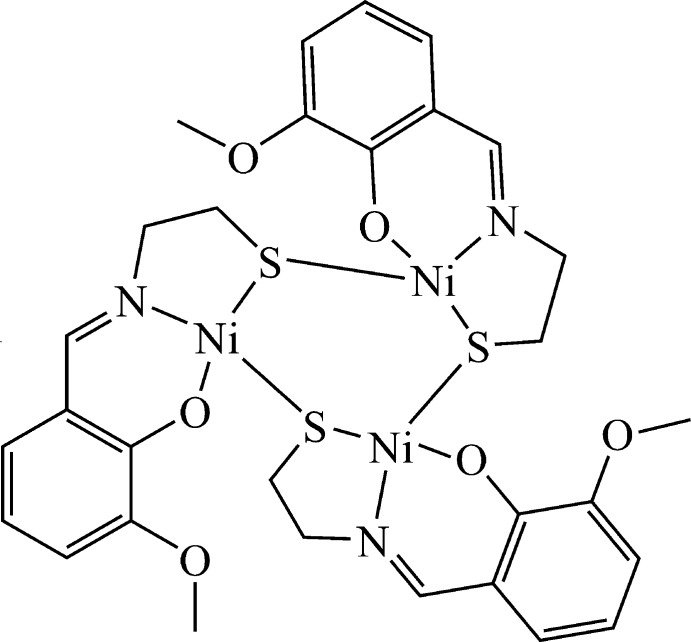


The coordination geometry around each Ni^II^ ion can be described as slightly distorted square planar and is comparable to that found in similar complexes reported previously (Kaasjager *et al.*, 2001 ▸; Constable *et al.*, 2011 ▸). Each Ni^II^ ion is tetra­coordinated by an identical NOS_2_ ligand environment: the dianionic Schiff base ligand occupies three of the four coordination sites (NOS), the fourth site place being filled by a bridging sulfur atom of a neighboring ligand. The deviation of the Ni^II^ atom from the NOS_2_ mean plane is 0.0927 (14) Å. Thus, the mol­ecule has a ‘crown’ or bowl shape with the Ni_3_S_3_ unit as its base in a distorted chair conformation. The torsion angles [between 78.49 (5) and 84.79 (5)°] deviate significantly from the ideal chair conformation for *c*-hexane which has torsion angles of 60°. For the Ni2 atom in this core, one additional short contact should be noted, C27—H27*C*⋯Ni(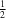 + *x*, *y*, 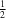 − *z*) with an H⋯Ni distance of 2.58 Å. Thus, with this additional contact, the coordination geometry of the Ni2 atom is square pyramidal with heteroatom—Ni2—H27*C* bond angles in the range 78.0-95.1°.

Unlike in closely related compounds, the solvent mol­ecule is not encapsulated. The distances observed between the Ni atoms are within Ni1⋯Ni2 3.5706 (4) and 3.6656 (5) Å. The intra­molecular Ni—S distances with the dianionic Schiff base ligand (Ni3—S4, Ni1—S5, Ni2—S6) are in the range 2.1888 (12)–2.2036 (13) Å. They are slightly shorter than analogous ones with the bridging sulfur atom (Ni3—S5, Ni1—S6, Ni2—S4) of the neighboring ligand [2.2171 (12)–2.2262 (13) Å]. These data are comparable with those previously reported for related structures (Kaasjager *et al.*, 2001 ▸; Henkel *et al.*, 1988 ▸).

## Supra­molecular features   

**Figure 2:**
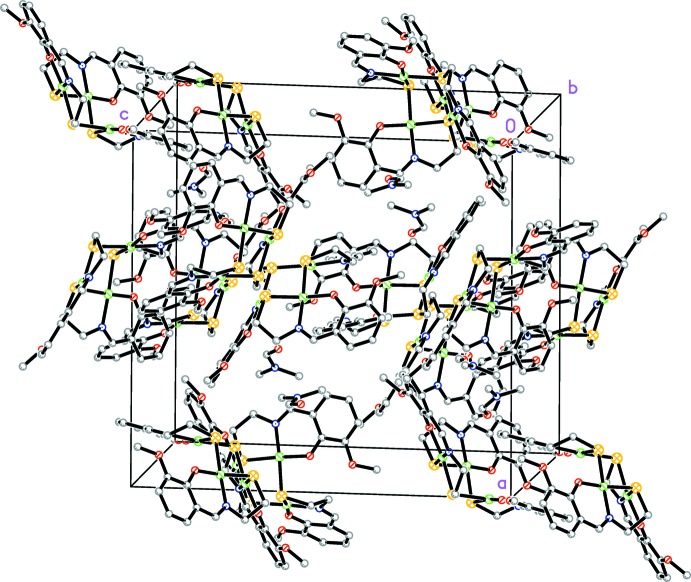
The crystal packing of the title compound. H atoms are not shown for clarity.

The solid-state organization of the complex can be described as an insertion of the solvent mol­ecules within the crystallographically independent trinuclear Ni^II^ species (Fig. 2 ▸).

In this structure, in contrast to previously reported analogous complexes (Constable *et al.*, 2011 ▸), the short C—H⋯Ni contact noted above connects neighboring structural units. This is slightly longer than analogous intra­molecular C—H⋯Ni contacts (2.21–2.40 Å; Stępień *et al.*, 2004 ▸; Gladkikh *et al.*, 2002 ▸) in metal–organic hydrides and hydro­boron-containing compounds. It seems that it is the first example of such a short inter­molecular C—H⋯Ni contact in coordination compounds.

**Figure 3:**
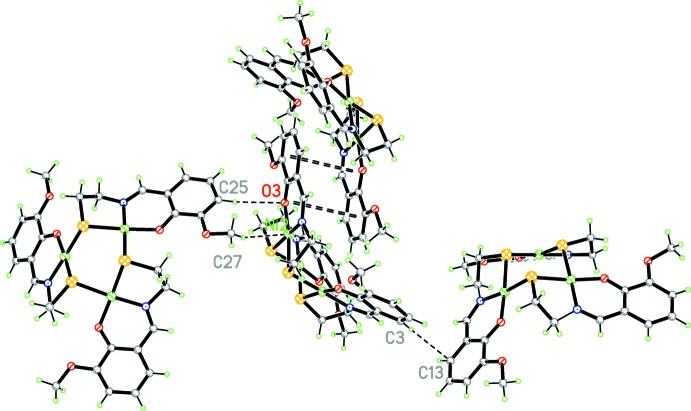
The crystal packing of the title compound. C—H⋯π edge-to-face inter­actions, π–π stacking inter­actions and the inter­molecular C—H⋯Ni contact that link the components in the crystal are shown as dashed lines.

In addition, π–π stacking inter­actions with a centroid–centroid distance *Cg*1⋯*Cg*2(−*x*, 2 − *y*,1 − *z*) of 3.722 (6) Å for connect the neighboring units (*Cg*1 and *Cg*2 are the centroids of the Ni2/O3/N3/C11/C12/C18 and C11–C16 rings, respectively). Several C—H⋯O and C—H⋯π edge-to-face inter­actions (Table 1 ▸) are also involved in linking the components in the crystal (Fig. 3 ▸).

## Database survey   

A search of the Cambridge Structural Database (Version 5.38; last update November 2016; Groom *et al.*, 2016 ▸) for related complexes with H⋯Ni contacts gave nine hits with intra­molecular *E*—H⋯Ni contacts where *E* is mainly Ir, Rh, B and only two examples with C—H⋯Ni. There were no *E*—H⋯Ni inter­molecular contacts found. A search for complexes with an Ni atom and an ONS Schiff base ligand gave 16 hits, including four closely related structures, *viz.* tris­{μ_2_-2-[(2-mercaptoeth­yl)imino­meth­yl]phenolato}trinickel and tris­(μ_2_-2-(2-naph­th­yl­meth­oxy)-6-[{(2-sulfido­eth­yl)imino]­meth­yl}phenolato)trinickel(II) di­chloro­methane solvate, tris­(μ_2_-2-(benz­yloxy)-6-{[(2-sulfido­eth­yl)imino]­meth­yl}phenolato)tri­nickel(II) di­chloro­methane solvate, tris­(μ_2_-2-eth­oxy-6-[{(2-sulfido­eth­yl)imino]­meth­yl}phenolato)trinickel(II) C60-fullerene dichloro­methane solvate, tris­(μ_2_-2-eth­oxy-6-{[(2-sulfido­eth­yl)imino]­meth­yl}phenolato)trinickel(II) di­chloro­methane solv­ate (Kaasjager *et al.*, 2001 ▸; Constable *et al.*, 2011 ▸).

## Synthesis and crystallization   

A solution of KOH (0.22 g, 4 mmol) in a minimum amount of methanol (2–3 ml) was added to a solution of 2-amino­ethane­thiol hydro­chloride (0.44g, 4 mmol) in methanol (5 ml) and stirred in an ice bath for 10 min. The white precipitate of solid KCl was removed by filtration and *o*-vaniline (0.61 g, 4 mmol) in ethanol (5 ml) was added to the filtrate and stirred on air magnetically for 2 h. Nickel acetate tetra­hydrate (0.99 g, 4 mmol) in ethanol (6 ml) was added to the yellowish solution of the Schiff base formed *in situ*, and the resulting deep-brown solution was stirred magnetically and heated at 340–347 K for 1.5 h resulting in a dark-colored precipitate. The product was isolated by filtration, washed with dry ^i=*i*^PrOH and finally dried *in vacuo*. Crystals suitable for crystallographic study were grown from a saturated solution in DMF (deep-brown solution). The crystals were filtered off, washed with dry *i*-PrOH and finally dried at room temperature (yield: 47%).

The IR spectrum of the title compound (as KBr pellets) is consistent with the above structural data. It displays the characteristic peak at 1610 cm^−1^ indicating the formation of a Schiff base (–H—C=N–) (Esteves-Souza *et al.*, 2006 ▸). The strong bands at 1330–1470 cm^−1^ can be attributed to overlapped C—H bending (scissoring) (as well in CH_3_ groups of the solvent mol­ecule) and aromatic –C=C– stretching vibrations. Other strong bands at 1228 and 1244 cm^−1^ are due to the phenolic CO stretching (Wu *et al.*, 2014 ▸). Two medium intensity bands observed at 620 and 738 cm^−1^ could be assigned to the asymmetric and symmetric C—S stretching vibrations, respectively. In the ^1^H-NMR spectrum, the azomethine proton peak that confirms the Schiff base formation is attributed to a singlet signal at 7.9 ppm. It overlaps with the solvent (DMF) proton signal. The O—CH_3_ protons peaks only appear at 3.92 ppm. The multiplets of the aromatic protons appear in the range 6.39–6.79 ppm with different multiplicity and coupling constants. The strong singlet at 3.39 ppm could be assigned to the aliphatic –CH_2_–CH_2_– protons according to its integral intensity. Signals from the DMF methyl protons appear at 2.94 and 2.78 ppm. Analysis calculated for for C_33_H_40_N_4_Ni_3_O_7_S_3_ (877.00): C, 45.20; H, 4.60; N, 6.39; found: C, 45.5; H, 4.77; N, 6.25.

## Refinement   

Crystal data, data collection and structure refinement details are summarized in Table 2 ▸. All hydrogen atoms were added at calculated positions (C—H = 0.93–0.97 Å) and refined using a riding model with *U*_iso_(H) = 1.2–1.5*U*_eq_(C).

## Anti­bacterial screening   

The anti­bacterial *in vitro* screening of all test compounds was carried out against reference strains of bacteria (American Type Culture Collection [ATCC] *Staphylococcus aureus* ATCC 25923, *Escherichia coli* ATCC 25922, *Pseudomonas aeruginosa* ATCC 27853) and clinical strains [*Acinetobacter baumannii* (MβL), *Klebsiella pneumoniae, Pseudomonas aeruginosa* (MβL), *Staphylococcus aureus* (MRCNS), *Staphylococcus aureus* (MRSA), *Staphylococcus aureus* (βL)]. The broth microdilution method was used according to the European Committee on Anti­microbial Susceptibility Testing (EUCAST). The results obtained indicate that the synthesized compound possesses a broad spectrum of activity against the tested microorganisms and shows relatively better activity against Gram-negative than Gram-positive bacteria. The title complex showed activity with lowest minimum inhibitory concentrations (MIC) values 312.5 µg ml^−1^ against Gram-negative bacteria *E. coli, K. pneumoniae* and *P. aeruginosa.* The highest activity was against clinical strain *A. baumannii* - MIC = 156.2 µg ml^−1^. The poorest activity of the complex was against clinical strain *Staphylococcus aureus* (MRSA). It is well known that *A. baumannii* is one of the most important nosocomial pathogens because of its longevity in the hospital environment and ability to resist various anti­microbial agents, such as resistance to broad-spectrum β-lactam anti­biotics by β-lactamases production (Peleg *et al.*, 2008 ▸; Jamulitrat *et al.*, 2007 ▸; Li *et al.*, 2007 ▸). The anti­bacterial study revealed that the title compound has anti­bacterial activity, the best being against *A. baumannii.*

## Supplementary Material

Crystal structure: contains datablock(s) I. DOI: 10.1107/S2056989019004730/ex2019sup1.cif

Structure factors: contains datablock(s) I. DOI: 10.1107/S2056989019004730/ex2019Isup2.hkl

CCDC reference: 1865532

Additional supporting information:  crystallographic information; 3D view; checkCIF report

## Figures and Tables

**Table 1:** Hydrogen-bond geometry (Å, °) *Cg*3 and *Cg*4 are the centroids of the C21–C26 and Ni1/O1/N1/C1/C2/C8, rings, respectively.

*D*—H⋯*A*	*D*—H	H⋯*A*	*D*⋯*A*	*D*—H⋯*A*
C18—H18⋯O7^i^	0.93	2.45	3.334 (6)	159
C29—H29*A*⋯S5^ii^	0.97	2.86	3.779 (5)	159
C25—H25⋯O3^iii^	0.93	2.71	3.6048 (4)	163
C3—H3⋯C13^iv^	0.93	2.85	3.7376 (4)	160
C5—H5⋯*Cg*3^v^	0.93	2.99	3.625 (5)	127
C15—H15⋯*Cg*4^vi^	0.93	2.83	3.604 (5)	142

Symmetry codes: (i) 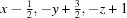; (ii) 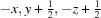; (iii) 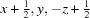; (iv) 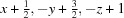; (v) 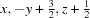; (vi) 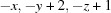.

**Table 2:** Experimental details

Crystal data	
Chemical formula	[Ni_3_(C_10_H_11_NO_2_S)_3_]·C_3_H_7_NO
*M*_r_	877.00
Crystal system, space group	Orthorhombic, *P* *b* *c* *a*
Temperature (K)	123
*a*, *b*, *c* (Å)	20.396 (3), 16.066 (3), 21.738 (3)
*V* (Å^3^)	7123.5 (19)
*Z*	8
Radiation type	Mo *K*α
μ (mm^−1^)	1.80
Crystal size (mm)	0.47 × 0.28 × 0.05

Data collection	
Diffractometer	Bruker *SMART* CCD area detector
Absorption correction	Multi-scan (*SADABS*; Bruker, 2008 ▸)
*T*_min_, *T*_max_	0.65, 0.92
No. of measured, independent and observed [*I* > 2σ(*I*)] reflections	49899, 6349, 4257
*R*_int_	0.107
(sin θ/λ)_max_ (Å^−1^)	0.597

Refinement	
*R*[*F* ^2^ > 2σ(*F* ^2^)], *wR*(*F* ^2^), *S*	0.042, 0.113, 1.00
No. of reflections	6349
No. of parameters	456
H-atom treatment	H-atom parameters constrained
Δρ_max_, Δρ_min_ (e Å^−3^)	0.57, −0.50

Computer programs: *SMART* and *SAINT* (Bruker, 2008 ▸), *SHELXT* (Sheldrick, 2015*a* ▸), *SHELXL2016/4* (Sheldrick, 2015*b* ▸), *SHELXTL* (Sheldrick, 2008 ▸) and *publCIF* (Westrip, 2010 ▸).

## supplementary crystallographic information

### Crystal data

**Table:**

[Ni_3_(C_10_H_11_NO_2_S)_3_]·C_3_H_7_NO	*D*_x_ = 1.635 Mg m^−^^3^
*M**_r_* = 877.00	Mo *K*α radiation, λ = 0.71073 Å
Orthorhombic, *P**b**c**a*	Cell parameters from 1664 reflections
*a* = 20.396 (3) Å	θ = 2.5–24.3°
*b* = 16.066 (3) Å	µ = 1.80 mm^−^^1^
*c* = 21.738 (3) Å	*T* = 123 K
*V* = 7123.5 (19) Å^3^	Plate, brown
*Z* = 8	0.47 × 0.28 × 0.05 mm
*F*(000) = 3632	

### Data collection

**Table:**

Bruker SMART CCD area detector diffractometer	6349 independent reflections
Radiation source: sealed tube	4257 reflections with *I* > 2σ(*I*)
Graphite monochromator	*R*_int_ = 0.107
phi and ω scans	θ_max_ = 25.1°, θ_min_ = 1.9°
Absorption correction: multi-scan (SADABS; Bruker, 2008)	*h* = −23→24
*T*_min_ = 0.65, *T*_max_ = 0.92	*k* = −19→19
49899 measured reflections	*l* = −25→25

### Refinement

**Table:**

Refinement on *F*^2^	0 restraints
Least-squares matrix: full	Hydrogen site location: inferred from neighbouring sites
*R*[*F*^2^ > 2σ(*F*^2^)] = 0.042	H-atom parameters constrained
*wR*(*F*^2^) = 0.113	*w* = 1/[σ^2^(*F*_o_^2^) + (0.0573*P*)^2^] where *P* = (*F*_o_^2^ + 2*F*_c_^2^)/3
*S* = 1.00	(Δ/σ)_max_ = 0.002
6349 reflections	Δρ_max_ = 0.57 e Å^−^^3^
456 parameters	Δρ_min_ = −0.50 e Å^−^^3^

### Special details

**Table:**

Geometry. All esds (except the esd in the dihedral angle between two l.s. planes) are estimated using the full covariance matrix. The cell esds are taken into account individually in the estimation of esds in distances, angles and torsion angles; correlations between esds in cell parameters are only used when they are defined by crystal symmetry. An approximate (isotropic) treatment of cell esds is used for estimating esds involving l.s. planes.

### Fractional atomic coordinates and isotropic or equivalent isotropic displacement parameters (Å^2^)

**Table:**

	*x*	*y*	*z*	*U*_iso_*/*U*_eq_	
C1	0.1040 (2)	0.6135 (3)	0.4547 (2)	0.0216 (11)	
N1	0.14902 (17)	0.6953 (2)	0.34331 (16)	0.0208 (9)	
NI1	0.05923 (3)	0.71688 (3)	0.35642 (2)	0.01757 (15)	
O1	0.05749 (14)	0.65749 (18)	0.42925 (13)	0.0218 (7)	
C2	0.1675 (2)	0.6052 (3)	0.4303 (2)	0.0245 (11)	
N2	0.05639 (17)	1.0287 (2)	0.27069 (15)	0.0175 (8)	
NI2	−0.05261 (3)	0.87936 (3)	0.39576 (2)	0.01647 (15)	
O2	0.13969 (15)	0.90693 (18)	0.23444 (14)	0.0262 (8)	
C3	0.2145 (2)	0.5541 (3)	0.4609 (2)	0.0314 (12)	
H3	0.256416	0.548700	0.444523	0.038*	
N3	−0.06396 (17)	0.8428 (2)	0.47725 (16)	0.0190 (8)	
NI3	0.05724 (3)	0.91137 (3)	0.26828 (2)	0.01665 (15)	
O3	−0.06788 (14)	0.98980 (17)	0.41482 (13)	0.0200 (7)	
C4	0.1988 (2)	0.5134 (3)	0.5132 (2)	0.0345 (13)	
H4	0.229727	0.479712	0.532412	0.041*	
N4	0.32425 (19)	0.8337 (3)	0.35718 (17)	0.0312 (10)	
O4	0.02740 (16)	0.58374 (18)	0.53216 (14)	0.0279 (8)	
S4	−0.04576 (5)	0.91131 (7)	0.29655 (5)	0.0179 (3)	
C5	0.1363 (2)	0.5220 (3)	0.5385 (2)	0.0293 (12)	
H5	0.126244	0.494494	0.574885	0.035*	
O5	0.23866 (15)	0.8613 (2)	0.16848 (15)	0.0331 (8)	
S5	0.05311 (5)	0.77300 (7)	0.26444 (5)	0.0190 (3)	
C6	0.0894 (2)	0.5703 (3)	0.5106 (2)	0.0260 (11)	
O6	−0.08955 (16)	1.15080 (19)	0.41606 (15)	0.0317 (8)	
S6	−0.04607 (5)	0.74826 (7)	0.36660 (5)	0.0193 (3)	
C7	0.0112 (3)	0.5505 (3)	0.5913 (2)	0.0361 (13)	
H7A	0.010203	0.490812	0.589061	0.054*	
H7B	−0.031052	0.570727	0.603684	0.054*	
H7C	0.043595	0.567557	0.620712	0.054*	
O7	0.35878 (19)	0.6998 (2)	0.34988 (15)	0.0476 (10)	
C8	0.1856 (2)	0.6482 (3)	0.3760 (2)	0.0235 (11)	
H8	0.228610	0.641402	0.362734	0.028*	
C9	0.1797 (2)	0.7362 (3)	0.28965 (19)	0.0236 (11)	
H9A	0.219139	0.706502	0.277862	0.028*	
H9B	0.191724	0.792901	0.300086	0.028*	
C10	0.1318 (2)	0.7364 (3)	0.2372 (2)	0.0244 (11)	
H10A	0.127330	0.680574	0.220734	0.029*	
H10B	0.147539	0.772455	0.204635	0.029*	
C11	−0.0862 (2)	1.0220 (3)	0.4676 (2)	0.0185 (10)	
C12	−0.0951 (2)	0.9762 (3)	0.5218 (2)	0.0212 (11)	
C13	−0.1154 (2)	1.0165 (3)	0.5762 (2)	0.0300 (12)	
H13	−0.120480	0.985565	0.612062	0.036*	
C14	−0.1277 (2)	1.0989 (3)	0.5774 (2)	0.0335 (13)	
H14	−0.141172	1.124339	0.613697	0.040*	
C15	−0.1201 (2)	1.1460 (3)	0.5236 (2)	0.0295 (12)	
H15	−0.128681	1.202832	0.524240	0.035*	
C16	−0.1002 (2)	1.1087 (3)	0.4703 (2)	0.0231 (11)	
C17	−0.1012 (3)	1.2377 (3)	0.4157 (2)	0.0403 (14)	
H17A	−0.146957	1.248147	0.422307	0.060*	
H17B	−0.088282	1.260377	0.376610	0.060*	
H17C	−0.076086	1.263611	0.447772	0.060*	
C18	−0.0820 (2)	0.8889 (3)	0.5231 (2)	0.0245 (11)	
H18	−0.086870	0.862371	0.560864	0.029*	
C19	−0.0501 (2)	0.7554 (3)	0.4905 (2)	0.0246 (11)	
H19A	−0.071544	0.739122	0.528505	0.030*	
H19B	−0.003255	0.747398	0.495504	0.030*	
C20	−0.0748 (2)	0.7026 (3)	0.43822 (19)	0.0235 (11)	
H20A	−0.122384	0.700919	0.438689	0.028*	
H20B	−0.058516	0.646188	0.442237	0.028*	
C21	0.1735 (2)	0.9672 (3)	0.21019 (19)	0.0192 (10)	
C22	0.1582 (2)	1.0516 (3)	0.21515 (19)	0.0196 (10)	
C23	0.1993 (2)	1.1128 (3)	0.1890 (2)	0.0267 (11)	
H23	0.188237	1.168805	0.192303	0.032*	
C24	0.2553 (2)	1.0900 (3)	0.1587 (2)	0.0269 (11)	
H24	0.283080	1.130485	0.142680	0.032*	
C25	0.2708 (2)	1.0059 (3)	0.1519 (2)	0.0263 (11)	
H25	0.308818	0.990638	0.131124	0.032*	
C26	0.2306 (2)	0.9456 (3)	0.1756 (2)	0.0211 (10)	
C27	0.2836 (2)	0.8347 (3)	0.1220 (2)	0.0356 (13)	
H27A	0.274122	0.863141	0.084227	0.053*	
H27B	0.279230	0.775766	0.115968	0.053*	
H27C	0.327566	0.847268	0.134711	0.053*	
C28	0.0997 (2)	1.0768 (3)	0.24669 (19)	0.0213 (11)	
H28	0.092408	1.133787	0.250102	0.026*	
C29	0.0019 (2)	1.0683 (3)	0.3039 (2)	0.0212 (11)	
H29A	−0.001822	1.126287	0.292097	0.025*	
H29B	0.009548	1.065625	0.347927	0.025*	
C30	−0.0598 (2)	1.0227 (3)	0.2879 (2)	0.0217 (10)	
H30A	−0.072285	1.035053	0.245854	0.026*	
H30B	−0.095042	1.040244	0.314861	0.026*	
C31	0.3514 (2)	0.7692 (4)	0.3282 (2)	0.0375 (13)	
H31	0.365864	0.777847	0.288189	0.045*	
C32	0.3015 (3)	0.8257 (3)	0.4199 (2)	0.0446 (15)	
H32A	0.307709	0.769365	0.433531	0.067*	
H32B	0.255822	0.839620	0.421934	0.067*	
H32C	0.326002	0.862642	0.445918	0.067*	
C33	0.3149 (3)	0.9136 (3)	0.3264 (3)	0.0478 (15)	
H33A	0.328520	0.909052	0.284238	0.072*	
H33B	0.340519	0.955447	0.346673	0.072*	
H33C	0.269354	0.928704	0.327920	0.072*	

### Atomic displacement parameters (Å^2^)

**Table:**

	*U*^11^	*U*^22^	*U*^33^	*U*^12^	*U*^13^	*U*^23^
C1	0.027 (3)	0.015 (2)	0.023 (3)	−0.001 (2)	−0.007 (2)	−0.005 (2)
N1	0.022 (2)	0.018 (2)	0.022 (2)	0.0000 (17)	0.0015 (17)	−0.0056 (17)
NI1	0.0168 (3)	0.0146 (3)	0.0214 (3)	0.0005 (2)	0.0007 (2)	−0.0002 (2)
O1	0.0237 (18)	0.0193 (17)	0.0224 (17)	0.0022 (14)	0.0009 (14)	0.0045 (14)
C2	0.026 (3)	0.016 (3)	0.031 (3)	0.002 (2)	−0.006 (2)	−0.007 (2)
N2	0.018 (2)	0.0160 (19)	0.019 (2)	−0.0005 (16)	0.0003 (17)	0.0000 (16)
NI2	0.0162 (3)	0.0149 (3)	0.0183 (3)	0.0006 (2)	0.0008 (2)	0.0001 (2)
O2	0.0262 (18)	0.0179 (18)	0.035 (2)	0.0023 (14)	0.0125 (15)	0.0070 (14)
C3	0.027 (3)	0.031 (3)	0.035 (3)	0.011 (2)	−0.011 (2)	−0.012 (2)
N3	0.017 (2)	0.020 (2)	0.020 (2)	0.0019 (16)	−0.0005 (17)	0.0029 (17)
NI3	0.0178 (3)	0.0135 (3)	0.0186 (3)	0.0000 (2)	0.0020 (3)	0.0009 (2)
O3	0.0264 (18)	0.0172 (17)	0.0164 (16)	0.0006 (13)	0.0012 (14)	−0.0031 (13)
C4	0.038 (3)	0.033 (3)	0.033 (3)	0.015 (2)	−0.015 (3)	0.000 (2)
N4	0.027 (2)	0.039 (3)	0.028 (2)	0.001 (2)	0.003 (2)	−0.006 (2)
O4	0.036 (2)	0.0204 (18)	0.0275 (19)	0.0015 (15)	0.0006 (16)	0.0066 (15)
S4	0.0174 (6)	0.0163 (6)	0.0198 (6)	0.0003 (5)	−0.0013 (5)	−0.0009 (5)
C5	0.045 (3)	0.018 (3)	0.025 (3)	−0.001 (2)	−0.014 (3)	0.003 (2)
O5	0.029 (2)	0.027 (2)	0.043 (2)	0.0035 (16)	0.0153 (17)	0.0023 (16)
S5	0.0216 (6)	0.0157 (6)	0.0199 (6)	−0.0013 (5)	0.0025 (5)	−0.0028 (5)
C6	0.029 (3)	0.018 (3)	0.030 (3)	0.000 (2)	−0.008 (2)	−0.004 (2)
O6	0.040 (2)	0.0171 (18)	0.038 (2)	0.0019 (16)	−0.0040 (17)	−0.0069 (15)
S6	0.0171 (6)	0.0156 (6)	0.0252 (6)	−0.0009 (5)	0.0012 (5)	−0.0004 (5)
C7	0.048 (3)	0.033 (3)	0.028 (3)	−0.002 (3)	0.007 (3)	0.006 (2)
O7	0.066 (3)	0.045 (3)	0.032 (2)	0.018 (2)	0.0031 (19)	−0.0013 (19)
C8	0.019 (3)	0.018 (3)	0.033 (3)	0.002 (2)	0.001 (2)	−0.005 (2)
C9	0.018 (2)	0.020 (3)	0.033 (3)	0.001 (2)	0.006 (2)	−0.005 (2)
C10	0.028 (3)	0.019 (3)	0.026 (3)	0.002 (2)	0.006 (2)	−0.004 (2)
C11	0.009 (2)	0.022 (3)	0.024 (3)	−0.0001 (19)	−0.005 (2)	−0.005 (2)
C12	0.014 (2)	0.027 (3)	0.022 (3)	0.002 (2)	−0.001 (2)	−0.006 (2)
C13	0.013 (2)	0.050 (4)	0.027 (3)	0.000 (2)	−0.005 (2)	−0.002 (2)
C14	0.025 (3)	0.042 (3)	0.034 (3)	0.008 (2)	−0.002 (2)	−0.023 (3)
C15	0.020 (3)	0.026 (3)	0.043 (3)	0.004 (2)	−0.004 (2)	−0.019 (2)
C16	0.020 (3)	0.024 (3)	0.026 (3)	0.002 (2)	−0.006 (2)	−0.005 (2)
C17	0.058 (4)	0.014 (3)	0.049 (3)	0.007 (2)	−0.022 (3)	−0.009 (2)
C18	0.014 (2)	0.038 (3)	0.022 (3)	−0.002 (2)	0.002 (2)	0.005 (2)
C19	0.023 (3)	0.022 (3)	0.029 (3)	0.006 (2)	0.006 (2)	0.009 (2)
C20	0.017 (2)	0.023 (3)	0.031 (3)	0.001 (2)	0.005 (2)	0.008 (2)
C21	0.013 (2)	0.029 (3)	0.016 (2)	−0.002 (2)	−0.001 (2)	0.006 (2)
C22	0.020 (3)	0.024 (3)	0.016 (2)	−0.003 (2)	−0.002 (2)	0.0027 (19)
C23	0.028 (3)	0.027 (3)	0.025 (3)	−0.008 (2)	0.000 (2)	0.001 (2)
C24	0.027 (3)	0.028 (3)	0.026 (3)	−0.012 (2)	−0.001 (2)	0.001 (2)
C25	0.020 (3)	0.036 (3)	0.023 (3)	−0.002 (2)	−0.003 (2)	−0.002 (2)
C26	0.018 (2)	0.021 (3)	0.025 (3)	0.001 (2)	−0.001 (2)	0.004 (2)
C27	0.023 (3)	0.037 (3)	0.047 (3)	0.004 (2)	0.011 (3)	0.005 (3)
C28	0.029 (3)	0.015 (2)	0.020 (2)	−0.003 (2)	−0.007 (2)	−0.0020 (19)
C29	0.026 (3)	0.013 (2)	0.024 (3)	0.0037 (19)	0.004 (2)	0.000 (2)
C30	0.023 (3)	0.021 (3)	0.021 (2)	0.007 (2)	−0.004 (2)	0.0076 (19)
C31	0.038 (3)	0.046 (4)	0.029 (3)	0.006 (3)	0.005 (3)	−0.003 (3)
C32	0.038 (3)	0.058 (4)	0.038 (3)	0.019 (3)	0.002 (3)	−0.002 (3)
C33	0.056 (4)	0.029 (3)	0.058 (4)	0.004 (3)	0.008 (3)	−0.005 (3)

### Geometric parameters (Å, º)

**Table:**

C1—O1	1.306 (5)	C9—H9A	0.9700
C1—C2	1.406 (6)	C9—H9B	0.9700
C1—C6	1.430 (6)	C10—H10A	0.9700
N1—C8	1.278 (5)	C10—H10B	0.9700
N1—C9	1.478 (5)	C11—C12	1.400 (6)
N1—Ni1	1.886 (4)	C11—C16	1.424 (6)
Ni1—O1	1.849 (3)	C12—C13	1.413 (6)
Ni1—S5	2.1970 (12)	C12—C18	1.428 (6)
Ni1—S6	2.2171 (12)	C13—C14	1.347 (6)
C2—C8	1.417 (6)	C13—H13	0.9300
C2—C3	1.426 (6)	C14—C15	1.401 (7)
N2—C28	1.285 (5)	C14—H14	0.9300
N2—C29	1.470 (5)	C15—C16	1.368 (6)
N2—Ni3	1.886 (3)	C15—H15	0.9300
Ni2—O3	1.849 (3)	C17—H17A	0.9600
Ni2—N3	1.881 (3)	C17—H17B	0.9600
Ni2—S6	2.2036 (13)	C17—H17C	0.9600
Ni2—S4	2.2213 (12)	C18—H18	0.9300
O2—C21	1.300 (5)	C19—C20	1.506 (6)
O2—Ni3	1.837 (3)	C19—H19A	0.9700
C3—C4	1.350 (7)	C19—H19B	0.9700
C3—H3	0.9300	C20—H20A	0.9700
N3—C18	1.295 (5)	C20—H20B	0.9700
N3—C19	1.461 (5)	C21—C22	1.395 (6)
Ni3—S4	2.1888 (12)	C21—C26	1.430 (6)
Ni3—S5	2.2262 (13)	C22—C23	1.412 (6)
O3—C11	1.313 (5)	C22—C28	1.435 (6)
C4—C5	1.395 (7)	C23—C24	1.369 (6)
C4—H4	0.9300	C23—H23	0.9300
N4—C31	1.333 (6)	C24—C25	1.395 (6)
N4—C32	1.446 (6)	C24—H24	0.9300
N4—C33	1.460 (6)	C25—C26	1.370 (6)
O4—C6	1.366 (5)	C25—H25	0.9300
O4—C7	1.430 (5)	C27—H27A	0.9600
S4—C30	1.821 (4)	C27—H27B	0.9600
C5—C6	1.373 (6)	C27—H27C	0.9600
C5—H5	0.9300	C28—H28	0.9300
O5—C26	1.372 (5)	C29—C30	1.496 (6)
O5—C27	1.429 (5)	C29—H29A	0.9700
S5—C10	1.808 (4)	C29—H29B	0.9700
O6—C16	1.376 (5)	C30—H30A	0.9700
O6—C17	1.417 (5)	C30—H30B	0.9700
S6—C20	1.818 (4)	C31—H31	0.9300
C7—H7A	0.9600	C32—H32A	0.9600
C7—H7B	0.9600	C32—H32B	0.9600
C7—H7C	0.9600	C32—H32C	0.9600
O7—C31	1.219 (6)	C33—H33A	0.9600
C8—H8	0.9300	C33—H33B	0.9600
C9—C10	1.502 (6)	C33—H33C	0.9600
			
O1—C1—C2	124.1 (4)	C13—C12—C18	119.2 (4)
O1—C1—C6	118.2 (4)	C14—C13—C12	121.4 (5)
C2—C1—C6	117.7 (4)	C14—C13—H13	119.3
C8—N1—C9	117.1 (4)	C12—C13—H13	119.3
C8—N1—Ni1	126.3 (3)	C13—C14—C15	119.6 (5)
C9—N1—Ni1	116.6 (3)	C13—C14—H14	120.2
O1—Ni1—N1	93.04 (14)	C15—C14—H14	120.2
O1—Ni1—S5	171.92 (10)	C16—C15—C14	120.3 (5)
N1—Ni1—S5	89.61 (11)	C16—C15—H15	119.9
O1—Ni1—S6	90.77 (10)	C14—C15—H15	119.9
N1—Ni1—S6	176.14 (12)	C15—C16—O6	123.9 (4)
S5—Ni1—S6	86.70 (4)	C15—C16—C11	121.5 (4)
C1—O1—Ni1	129.0 (3)	O6—C16—C11	114.5 (4)
C1—C2—C8	120.5 (4)	O6—C17—H17A	109.5
C1—C2—C3	119.9 (4)	O6—C17—H17B	109.5
C8—C2—C3	119.6 (4)	H17A—C17—H17B	109.5
C28—N2—C29	117.4 (4)	O6—C17—H17C	109.5
C28—N2—Ni3	125.7 (3)	H17A—C17—H17C	109.5
C29—N2—Ni3	116.9 (3)	H17B—C17—H17C	109.5
O3—Ni2—N3	93.92 (14)	N3—C18—C12	126.8 (4)
O3—Ni2—S6	172.82 (9)	N3—C18—H18	116.6
N3—Ni2—S6	88.83 (11)	C12—C18—H18	116.6
O3—Ni2—S4	90.36 (9)	N3—C19—C20	109.1 (4)
N3—Ni2—S4	173.96 (11)	N3—C19—H19A	109.9
S6—Ni2—S4	86.43 (4)	C20—C19—H19A	109.9
C21—O2—Ni3	128.2 (3)	N3—C19—H19B	109.9
C4—C3—C2	120.8 (5)	C20—C19—H19B	109.9
C4—C3—H3	119.6	H19A—C19—H19B	108.3
C2—C3—H3	119.6	C19—C20—S6	108.2 (3)
C18—N3—C19	116.9 (4)	C19—C20—H20A	110.1
C18—N3—Ni2	125.5 (3)	S6—C20—H20A	110.1
C19—N3—Ni2	117.6 (3)	C19—C20—H20B	110.1
O2—Ni3—N2	93.34 (14)	S6—C20—H20B	110.1
O2—Ni3—S4	172.34 (10)	H20A—C20—H20B	108.4
N2—Ni3—S4	89.08 (11)	O2—C21—C22	125.0 (4)
O2—Ni3—S5	88.90 (10)	O2—C21—C26	117.6 (4)
N2—Ni3—S5	177.25 (11)	C22—C21—C26	117.3 (4)
S4—Ni3—S5	88.50 (4)	C21—C22—C23	120.9 (4)
C11—O3—Ni2	128.4 (3)	C21—C22—C28	119.8 (4)
C3—C4—C5	120.0 (4)	C23—C22—C28	119.3 (4)
C3—C4—H4	120.0	C24—C23—C22	120.2 (4)
C5—C4—H4	120.0	C24—C23—H23	119.9
C31—N4—C32	120.6 (4)	C22—C23—H23	119.9
C31—N4—C33	121.4 (4)	C23—C24—C25	120.0 (4)
C32—N4—C33	118.0 (4)	C23—C24—H24	120.0
C6—O4—C7	117.6 (4)	C25—C24—H24	120.0
C30—S4—Ni3	96.96 (15)	C26—C25—C24	120.6 (4)
C30—S4—Ni2	108.50 (15)	C26—C25—H25	119.7
Ni3—S4—Ni2	109.46 (5)	C24—C25—H25	119.7
C6—C5—C4	121.3 (5)	C25—C26—O5	125.7 (4)
C6—C5—H5	119.4	C25—C26—C21	120.9 (4)
C4—C5—H5	119.4	O5—C26—C21	113.4 (4)
C26—O5—C27	116.9 (3)	O5—C27—H27A	109.5
C10—S5—Ni1	96.54 (15)	O5—C27—H27B	109.5
C10—S5—Ni3	107.67 (15)	H27A—C27—H27B	109.5
Ni1—S5—Ni3	111.94 (5)	O5—C27—H27C	109.5
O4—C6—C5	125.7 (4)	H27A—C27—H27C	109.5
O4—C6—C1	114.0 (4)	H27B—C27—H27C	109.5
C5—C6—C1	120.3 (5)	N2—C28—C22	126.6 (4)
C16—O6—C17	117.6 (4)	N2—C28—H28	116.7
C20—S6—Ni2	96.86 (15)	C22—C28—H28	116.7
C20—S6—Ni1	107.85 (15)	N2—C29—C30	108.0 (4)
Ni2—S6—Ni1	107.75 (5)	N2—C29—H29A	110.1
O4—C7—H7A	109.5	C30—C29—H29A	110.1
O4—C7—H7B	109.5	N2—C29—H29B	110.1
H7A—C7—H7B	109.5	C30—C29—H29B	110.1
O4—C7—H7C	109.5	H29A—C29—H29B	108.4
H7A—C7—H7C	109.5	C29—C30—S4	109.0 (3)
H7B—C7—H7C	109.5	C29—C30—H30A	109.9
N1—C8—C2	126.9 (4)	S4—C30—H30A	109.9
N1—C8—H8	116.6	C29—C30—H30B	109.9
C2—C8—H8	116.6	S4—C30—H30B	109.9
N1—C9—C10	108.9 (3)	H30A—C30—H30B	108.3
N1—C9—H9A	109.9	O7—C31—N4	125.5 (5)
C10—C9—H9A	109.9	O7—C31—H31	117.3
N1—C9—H9B	109.9	N4—C31—H31	117.3
C10—C9—H9B	109.9	N4—C32—H32A	109.5
H9A—C9—H9B	108.3	N4—C32—H32B	109.5
C9—C10—S5	109.3 (3)	H32A—C32—H32B	109.5
C9—C10—H10A	109.8	N4—C32—H32C	109.5
S5—C10—H10A	109.8	H32A—C32—H32C	109.5
C9—C10—H10B	109.8	H32B—C32—H32C	109.5
S5—C10—H10B	109.8	N4—C33—H33A	109.5
H10A—C10—H10B	108.3	N4—C33—H33B	109.5
O3—C11—C12	124.4 (4)	H33A—C33—H33B	109.5
O3—C11—C16	118.5 (4)	N4—C33—H33C	109.5
C12—C11—C16	117.1 (4)	H33A—C33—H33C	109.5
C11—C12—C13	120.1 (4)	H33B—C33—H33C	109.5
C11—C12—C18	120.6 (4)		
			
C8—N1—Ni1—O1	5.2 (4)	N3—Ni2—S6—Ni1	−98.96 (11)
C9—N1—Ni1—O1	−175.5 (3)	S4—Ni2—S6—Ni1	84.79 (5)
C8—N1—Ni1—S5	−167.2 (4)	O1—Ni1—S6—C20	1.12 (18)
C9—N1—Ni1—S5	12.2 (3)	N1—Ni1—S6—C20	−169.8 (17)
C8—N1—Ni1—S6	176.1 (14)	S5—Ni1—S6—C20	173.45 (16)
C9—N1—Ni1—S6	−4.5 (19)	O1—Ni1—S6—Ni2	104.70 (10)
C2—C1—O1—Ni1	1.8 (6)	N1—Ni1—S6—Ni2	−66.3 (17)
C6—C1—O1—Ni1	−177.5 (3)	S5—Ni1—S6—Ni2	−82.97 (5)
N1—Ni1—O1—C1	−4.6 (4)	C9—N1—C8—C2	177.7 (4)
S5—Ni1—O1—C1	104.4 (7)	Ni1—N1—C8—C2	−3.0 (7)
S6—Ni1—O1—C1	176.0 (3)	C1—C2—C8—N1	−1.7 (7)
O1—C1—C2—C8	2.4 (7)	C3—C2—C8—N1	179.2 (4)
C6—C1—C2—C8	−178.3 (4)	C8—N1—C9—C10	141.1 (4)
O1—C1—C2—C3	−178.5 (4)	Ni1—N1—C9—C10	−38.3 (4)
C6—C1—C2—C3	0.8 (6)	N1—C9—C10—S5	48.1 (4)
C1—C2—C3—C4	−0.1 (7)	Ni1—S5—C10—C9	−35.2 (3)
C8—C2—C3—C4	179.0 (4)	Ni3—S5—C10—C9	80.3 (3)
O3—Ni2—N3—C18	4.9 (4)	Ni2—O3—C11—C12	2.9 (6)
S6—Ni2—N3—C18	−168.4 (4)	Ni2—O3—C11—C16	−175.0 (3)
S4—Ni2—N3—C18	−130.1 (10)	O3—C11—C12—C13	−179.4 (4)
O3—Ni2—N3—C19	−173.7 (3)	C16—C11—C12—C13	−1.6 (6)
S6—Ni2—N3—C19	12.9 (3)	O3—C11—C12—C18	2.3 (7)
S4—Ni2—N3—C19	51.3 (12)	C16—C11—C12—C18	−179.8 (4)
C21—O2—Ni3—N2	−12.9 (4)	C11—C12—C13—C14	1.0 (7)
C21—O2—Ni3—S4	95.3 (8)	C18—C12—C13—C14	179.3 (4)
C21—O2—Ni3—S5	165.5 (3)	C12—C13—C14—C15	−0.1 (7)
C28—N2—Ni3—O2	7.8 (4)	C13—C14—C15—C16	−0.2 (7)
C29—N2—Ni3—O2	−171.1 (3)	C14—C15—C16—O6	−177.9 (4)
C28—N2—Ni3—S4	−164.9 (3)	C14—C15—C16—C11	−0.4 (7)
C29—N2—Ni3—S4	16.2 (3)	C17—O6—C16—C15	−1.1 (6)
C28—N2—Ni3—S5	−137 (2)	C17—O6—C16—C11	−178.7 (4)
C29—N2—Ni3—S5	44 (2)	O3—C11—C16—C15	179.3 (4)
N3—Ni2—O3—C11	−5.6 (3)	C12—C11—C16—C15	1.3 (6)
S6—Ni2—O3—C11	106.8 (8)	O3—C11—C16—O6	−3.0 (6)
S4—Ni2—O3—C11	170.2 (3)	C12—C11—C16—O6	179.0 (4)
C2—C3—C4—C5	−0.8 (7)	C19—N3—C18—C12	176.9 (4)
O2—Ni3—S4—C30	−98.9 (8)	Ni2—N3—C18—C12	−1.8 (7)
N2—Ni3—S4—C30	9.66 (17)	C11—C12—C18—N3	−2.8 (7)
S5—Ni3—S4—C30	−169.04 (14)	C13—C12—C18—N3	178.9 (4)
O2—Ni3—S4—Ni2	148.7 (7)	C18—N3—C19—C20	141.6 (4)
N2—Ni3—S4—Ni2	−102.80 (11)	Ni2—N3—C19—C20	−39.6 (4)
S5—Ni3—S4—Ni2	78.49 (5)	N3—C19—C20—S6	48.4 (4)
O3—Ni2—S4—C30	−1.07 (17)	Ni2—S6—C20—C19	−35.0 (3)
N3—Ni2—S4—C30	134.1 (11)	Ni1—S6—C20—C19	76.1 (3)
S6—Ni2—S4—C30	172.50 (15)	Ni3—O2—C21—C22	12.5 (6)
O3—Ni2—S4—Ni3	103.62 (10)	Ni3—O2—C21—C26	−167.4 (3)
N3—Ni2—S4—Ni3	−121.2 (11)	O2—C21—C22—C23	177.7 (4)
S6—Ni2—S4—Ni3	−82.81 (5)	C26—C21—C22—C23	−2.5 (6)
C3—C4—C5—C6	1.0 (7)	O2—C21—C22—C28	−2.9 (7)
O1—Ni1—S5—C10	−96.8 (7)	C26—C21—C22—C28	176.9 (4)
N1—Ni1—S5—C10	12.42 (18)	C21—C22—C23—C24	−0.6 (7)
S6—Ni1—S5—C10	−168.69 (15)	C28—C22—C23—C24	180.0 (4)
O1—Ni1—S5—Ni3	151.1 (7)	C22—C23—C24—C25	2.1 (7)
N1—Ni1—S5—Ni3	−99.64 (11)	C23—C24—C25—C26	−0.4 (7)
S6—Ni1—S5—Ni3	79.25 (5)	C24—C25—C26—O5	175.7 (4)
O2—Ni3—S5—C10	4.21 (18)	C24—C25—C26—C21	−2.9 (7)
N2—Ni3—S5—C10	149 (2)	C27—O5—C26—C25	−14.6 (6)
S4—Ni3—S5—C10	177.01 (15)	C27—O5—C26—C21	164.2 (4)
O2—Ni3—S5—Ni1	109.11 (11)	O2—C21—C26—C25	−175.9 (4)
N2—Ni3—S5—Ni1	−106 (2)	C22—C21—C26—C25	4.3 (6)
S4—Ni3—S5—Ni1	−78.09 (5)	O2—C21—C26—O5	5.3 (6)
C7—O4—C6—C5	5.5 (6)	C22—C21—C26—O5	−174.5 (4)
C7—O4—C6—C1	−173.1 (4)	C29—N2—C28—C22	176.7 (4)
C4—C5—C6—O4	−178.8 (4)	Ni3—N2—C28—C22	−2.2 (6)
C4—C5—C6—C1	−0.3 (7)	C21—C22—C28—N2	−2.2 (7)
O1—C1—C6—O4	−2.6 (6)	C23—C22—C28—N2	177.2 (4)
C2—C1—C6—O4	178.1 (4)	C28—N2—C29—C30	138.8 (4)
O1—C1—C6—C5	178.7 (4)	Ni3—N2—C29—C30	−42.2 (4)
C2—C1—C6—C5	−0.6 (6)	N2—C29—C30—S4	48.7 (4)
O3—Ni2—S6—C20	−100.3 (8)	Ni3—S4—C30—C29	−33.7 (3)
N3—Ni2—S6—C20	12.30 (18)	Ni2—S4—C30—C29	79.5 (3)
S4—Ni2—S6—C20	−163.95 (15)	C32—N4—C31—O7	1.1 (8)
O3—Ni2—S6—Ni1	148.4 (8)	C33—N4—C31—O7	−177.2 (5)

### Hydrogen-bond geometry (Å, º)

*Cg*3 and *Cg*4 are the centroids of the C21–C26 and Ni1/O1/N1/C1/C2/C8, rings, respectively.

**Table:**

*D*—H···*A*	*D*—H	H···*A*	*D*···*A*	*D*—H···*A*
C18—H18···O7^i^	0.93	2.45	3.334 (6)	159
C29—H29*A*···S5^ii^	0.97	2.86	3.779 (5)	159
C25—H25···O3^iii^	0.93	2.71	3.6048 (4)	163
C3—H3···C13^iv^	0.93	2.85	3.7376 (4)	160
C5—H5···*Cg*3^v^	0.93	2.99	3.625 (5)	127
C15—H15···*Cg*4^vi^	0.93	2.83	3.604 (5)	142

Symmetry codes: (i) *x*−1/2, −*y*+3/2, −*z*+1; (ii) −*x*, *y*+1/2, −*z*+1/2; (iii) *x*+1/2, *y*, −*z*+1/2; (iv) *x*+1/2, −*y*+3/2, −*z*+1; (v) *x*, −*y*+3/2, *z*+1/2; (vi) −*x*, −*y*+2, −*z*+1.
